# Causality Orientations and Supportive/Controlled Environment: Understanding Their Influence on Basic Needs, Motivation for Health and Emotions in French Hospitalized Older Adults

**DOI:** 10.3389/fpsyg.2020.575489

**Published:** 2020-12-09

**Authors:** Guillaume Souesme, Guillaume Martinent, Donia Akour, Caroline Giraudeau, Claude Ferrand

**Affiliations:** ^1^Laboratoire de Psychologie des Âges de la Vie et Adaptation (PAVéA-EA 2114), Université de Tours, Tours, France; ^2^Laboratoire sur les Vulnérabilités et l’Innovation dans le Sport (L-Vis-EA 7428), Université Lyon 1, Villeurbanne, France; ^3^Psychologist, Tours, France

**Keywords:** motivation, causality orientations, social environment, basic needs, older adults

## Abstract

**Objectives:**

From a self-determination theory perspective, the purpose of this cross sectional study was to better understand how to motivate hospitalized older adults’ behaviors and test an integrative model of the role of causality orientations and a supportive/controlled environment on basic need satisfaction, motivation for health oriented physical activity, positive and negative affective states, depressive symptoms, apathy, and boredom.

**Methods:**

Older adults (*N* = 146; *M_age_* = 81.27 years, *SD* = 7.75, 74.7% female) in French hospital units completed self-report questionnaires and socio-demographic data were also collected.

**Results:**

Partial least squares path modeling results showed that participants’ autonomy orientation positively associated with the perception of a supportive environment was related to need satisfaction, autonomous motivation for health-oriented physical activity, and high scores on positive affective states. Conversely, participants’ impersonal orientation positively associated with the perception of a controlled environment was related to undermining need satisfaction, controlled motivation for health-oriented physical activity, and amotivation, and high scores on both negative affective states, and boredom.

**Conclusion:**

The present results indicate that taking into account personality factors in hospital units can be useful and health professionals should take an interest both in the patients’ causality orientations and the supportive nature of the environment in order to understand better how to motivate patients’ behaviors. The present study points to the need to focus on all motivational dynamics which allow patients’ need satisfaction.

## Introduction

Older people are a frail population often in high proportion in hospital units. In France, certain structures exist such as after-care and rehabilitation services (A-CRS) which have the dual aim of ensuring older people’s access to appropriate care and optimizing management of hospital stays. Within these units, the average length of stay is 34.8 days (Coquelet and Valdelièvre, 2011), with a return home for 82% of patients, placement in medical-surgery health units for 13%, and admission into a nursing home for 5% (Coquelet, 2015). Hospitalization time is an important issue because if repeated and/or prolonged, it may produce negative consequences for older patients’ health (e.g., Berkman et al., 2001). Consequently, the aims of A-CRS are (1) to provide medical care and rehabilitation programs in order to decrease physical, cognitive, and behavioral impairments; and (2) to prevent the onset of dependence and promote the patient’s functional autonomy. Adherence to rehabilitation programs is a key aspect in the success of these units and motivation for health oriented physical activity greatly influences behavior outcomes (Boiché et al., 2016). Ryan and Deci (2017) argued that the level and nature of motivation (autonomous vs. controlled) depended on the need satisfaction. So, need-supportive programs to physical activity have been shown to improve patients’ quality of motivation and to influence long-term physical activity adherence (e.g., Rodrigues et al., 2018), and a collaborative and empathetic approach from health professionals to foster patients’ psychological need satisfaction (e.g., Lock et al., 2018).

The present study is based on the self-determination theory (SDT) an “organismic theory of human motivation and personality development” which approaches psychological growth, integrity, and wellness as a life science (Ryan and Deci, 2017, p. 3), and comprises several mini-theories that were developed to explain different motivational processes. Central to SDT (Ryan and Deci, 2000), are claims that human growth and activity potentials are achieved through satisfaction of basic psychological needs for autonomy (need to be the perceived at the origin of one’s own behavior), competence (need to feel effective and have the opportunity to exercise one’s capacities) and relatedness (need to feel connected to others to care for and be cared for by others) considered as essential nutrients necessary for optimal psychological functioning and perceived concordance between personal agency and action (Ryan and Deci, 2000). The Basic Psychological Needs Theory attempts to explain the relationship between need satisfaction and motivation and supports the hypothesis that satisfaction of basic psychological needs affects the quality of one’s motivation and is a strong predictor of autonomous motivation (e.g., Kirkland et al., 2011). Moreover, the Cognitive Evaluation Theory emphasizes contextual determinants which play a crucial role in supporting or thwarting these three needs and offers a way of understanding what people really need from their psychological and social environments to function fully and to thrive. SDT indicates that people could be differentially motivated by different social conditions and highlights the role of interpersonal behaviors in influencing the environment through their communication style and activities. Thus, the content and nature of communications are key determinants of whether an environment is perceived as supportive or controlling. More specifically, supportive socializers create opportunities for individuals to take initiatives, develop goals and provide choices, relevant informational feedback, and challenges that enable individuals to use their skills, share decision-making, and encourage connectedness with others and supportive social relationships. Controlled socializers pressure individuals to act, think, or feel in prescribed ways and have cold interactions with them (Deci and Ryan, 2000). Several studies have shown that a need-supportive environment is linked to various positive outcomes for patients (Ng et al., 2012), such as better dental hygiene (Halvari et al., 2017), lower scores on depressive symptoms and apathy (Souesme et al., 2016), a better quality of life for haemodialysis patients (Chen et al., 2017), a greater responsibility in the management of diabetes and glycemic control (Lee et al., 2019) and a better persistence and adherence to rehabilitation programs (Scholz et al., 2006; Chan et al., 2009). However, need satisfaction does not only depend on a supportive environment. Deci and Ryan (1985) indicated that the dispositional motivational orientations of an individual may also have an influence on the way in which the situation is perceived as ambiguous or not. These dispositional tendencies allow to interpret the environment as more or less supportive of need satisfaction (Deci and Ryan, 2000). Some people have “a greater capacity to experience events as a source of information for initiating and regulating their own chosen behaviors and to maintain a higher level of autonomous motivation regardless of the objective properties of the event” (Deci and Ryan, 1985, p. 110). Others tend, to a greater degree, to look for existing controls in the environment. They show little autonomous motivation, although they may become very competent once they have learned operational contingencies or rules. Still others are quite easily shaken in their perception of competence and self-awareness. They tend to experience a wide range of events as amotivating. These three types of dispositional motivational tendencies have been developed in the Causality Orientations Theory and intituled causality orientations “to better convey the idea of orientations toward the initiation and regulation of behavior” (Deci and Ryan, 1985, p 111). Olesen (2011) suggested that autonomy orientation involves a high degree of choice experienced as free and volitional in accordance with one’s own standards and beliefs. Control orientation implies that people’s behavior should be organized with respect to control either in the environment or inside themselves. Impersonal causality orientation is focused on performance anxieties and on avoiding failure. Individuals tend to believe that they are unable to regulate their behavior in a way that will reliably lead to desired outcomes. Each orientation can be differentially salient to an individual, often as a function of context. However, Deci and Ryan (1985, p. 111–112) showed that (1) “a strong autonomy orientation leads people to select jobs that allow greater initiative, to interpret their existing situations as more autonomy promoting, i.e., as informational), and to organize their actions on the basis of personal goals and interests rather than controls and constraints”; (2) “a strong control orientation leads people to tend to do something because they think ‘they should’ and to rely on controlling events such as surveillance to motivate themselves”; and (3) “a strong impersonal orientation leads people to see themselves as incompetent and unable to master situations.”

**FIGURE 1:**
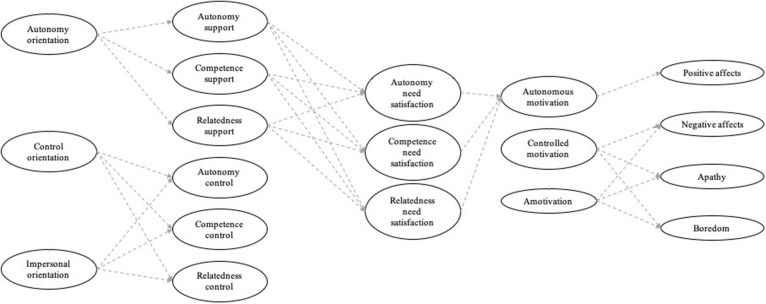
Hypothesised structural model of the partial least square path modeling.

This reflection seems interesting insofar as analyzing motivational phenomena from the perspective of people’s orientations toward the initiation and regulation of behavior can improve our understanding of the behaviors of hospitalized older people. Given that Hagger and Chatzisarantis (2011) showed that causality orientations could interact with contextual determinants in influencing need satisfaction and motivation, the present study seeks to explore the complete sequence of relationships between causality orientations, a supportive/controlled environment, basic need satisfaction, motivation for health-oriented physical activity, and emotions. Emotions have an important relationship with motivation for health oriented physical activity operating as feedback to reinforce or not the behavior (Kwan and Bryan, 2010). Examining this complete sequence is important, not only for theorists but also for practitioners wishing to improve both the quality of patients’ stays and their adherence to rehabilitation and also to prevent older patients returning to hospital. Based on SDT research, the following four hypotheses were formulated: (a) H_0a_: autonomy orientation would be positively associated with a perception of autonomy support, and controlled and impersonal orientations would be positively associated with a perception of controlled support; (b) H_0b_: autonomy support would be positively associated with basic need satisfaction; (c) H_0c_: basic need satisfaction would be positively associated with autonomous motivation for health oriented physical activity; (d) H_0d_: autonomous motivation for health oriented physical activity would be positively associated with positive affective states, and controlled motivation or amotivation for health oriented physical activity would be positively associated with negative affective states, apathy, and boredom. See Figure 1.

## Materials and Methods

### Structure

Six A-CRS with a total of 403 beds agreed to participate in this study. All the management bodies accepted to participate following one or two meetings conducted by the first author to explain the interest and aim of the study. Verbal consent was then obtained from medical teams and management before the beginning of the study. Although four A-CRS were public and two were private, there were no differences in terms of their running or services proposed to patients. Indeed, in France, no matter the sector of the industry, the objective of health services is determined by national law. In this way, they should propose the same care to patients. Table 1 shows the characteristics of A-CRS.

**TABLE 1:** Characteristics of A-CRS.

***N***	**A-CRS #1**	**A-CRS #2**	**A-CRS #3**	**A-CRS #4**	**A-CRS #5**	**A-CRS #6**
Staff	57	27	35	68	47	50
Beds	96	36	46	105	60	60
Industry	Public	Public	Public	Private	Public	Private
Average stays length (days)	49.5	36	38	34.6	41	40
Patients interviewed	6	4	3	110	3	20

*A-CRS, After-care and rehabilitation services.*

### Participants

The inclusion criteria for determining our population were as follows: 65 years old and over, no history of cyclical depression, and no severe cognitive impairment. To exclude individuals with severe cognitive impairment, we used the French version (Desrosiers and Hébert, 1997) of the Mini Mental State Examination (MMSE). Individuals with an MMSE score lower than 24 were excluded from the study, as they were likely to have difficulty understanding the questionnaires. A recruitment population of 200 patients/403 was possible.

One hundred and eighty-four patients agreed to participate in the study and data collection was conducted between January and July 2017. One hundred and forty-six older adults (109 women, 37 men, *Mage* = 81.27 years, *SD* = 7.75, age range: 65–99 years) completed all measures, resulting in a response rate of 79%. The average length of a stay was around 37 days, and no significant differences were observed between A-CRS length of stay. Table 2 shows the distribution of socio-demographic variables.

**TABLE 2:** Distribution of sociodemographic variables.

**Variables**	***N***	**Variables**	***N***
**Gender**		**Environment of the place of residence**	
Men	37	Rural area	41
Women	109	Urban area	69
**Marital status**		Semi-urban area	36
Single	17	**Chronic illness**	
Married	42	Yes	59
Widowed	72	No	87
Separated/divorced	15	**Iso-resources group (GIR)**	
**Place of residence**		1	77
Home	139	2	47
Nursing home	1	3	19
At a child’s house	1	4	3
Other	5	**Post hospitalization plan**	
**Past professional occupation**		Home	124
Farmers	8	Nursing home	14
Artisans/shopkeepers	26	Other	8
Middle executives	17		
Top executives	5		
Employees	57		
Unskilled/semiskilled workers	16		
Without professional activity	17		
**Level of education**			
No diploma	26		
Primary school	55		
High school	50		
University or equivalent	15		

*N = 146. GIR1, A person who is still autonomous for essential activities of everyday life; GIR 2, A person who only needs occasional help with washing, meal preparation and cleaning; GIR 3, A person who cannot get up alone but who, once up, can move about inside his/her accommodation, and who needs help for washing and dressing; GIR 4, A person who has retained his/her mental autonomy/faculties, who has partial locomotor autonomy/mobility, but who needs daily help for personal care and everyday activities.*

### Materials

#### Causality Orientations

The General Causality Orientations Scale was used to asses causality orientations (Vallerand et al., 1987). Organizational items were adapted to match the patients’ environment. The questions covered the rehabilitation activities organized during their stay and behaviors associated with hospitalized older people. For each of the seven questions, patients were given three options related to the three causality orientations described by Deci and Ryan (1985). Cronbach alphas in this study were 0.61, 0.43, and 0.72 for autonomy, controlled and impersonal orientations, respectively. Participants were asked to rate their agreement with each statement using a scale ranging from 1 (very unlikely) to 7 (very likely).

#### Supportive vs. Controlled Environment

We used the Interpersonal Behavior Questionnaire (Rocchi et al., 2017). Items referring to interpersonal behaviors in sport were adjusted to our participants’ environment. This scale is composed of 24 items, including four items referring to autonomy support (e.g., “I feel that health professionals give me the freedom to make my own choices”), four items evaluating controlled autonomy (e.g., “Health professionals put pressure on me to do everyday activities their way”), four items measuring competence support (e.g., “Health professionals encourage me to improve my abilities”), four items referring to controlled competence (e.g., “Health professionals point out that I will probably fail in everyday tasks.”), four items rating relatedness support (e.g., “Health professionals are interested in things that I do every day.”), and four items measuring controlled relatedness (e.g., “Health professionals do not comfort me when I feel low”). Participants were asked to rate their agreement with each statement using a scale ranging from 1 (strongly disagree) to 7 (strongly agree). Cronbach alphas were 0.78, 0.67, 0.76 for autonomy, competence and relatedness support and 0.82, 0.83, and 0.11 for controlled autonomy, competence, and relatedness, respectively.

#### Basic Psychological Needs

Three questionnaires were used to assess the perception of each psychological need on a scale ranging from 1 (strongly disagree) to 5 (strongly agree). We used (1) five items of the Standage et al.’s (2003) questionnaire (e.g., “I feel free to express ideas and opinions”) to assess autonomy need satisfaction; (2) five items of the perceived competence subscale of the Intrinsic Motivation Inventory (McAuley et al., 1989, e.g., “I often feel very competent”) to assess competence need satisfaction, and (3) six items of the Need for Relatedness Scale (Richer and Vallerand, 1998, e.g., “It is important for me to be close to others”) to assess relatedness need satisfaction. All items were preceded by the phrase “In this care unit….,” in order to help participants to respond to these questionnaires. In this study, Cronbach’s alphas for satisfaction of need for autonomy, competence and relatedness were 0.79, 0.72, and 0.77, respectively, showing acceptable internal consistency.

#### Motivation for Health Oriented Physical Activity

We used the French version of Motivation for Health-Oriented Physical Activity Scale (Boiché et al., 2016). Autonomous motivation score was calculated including three items measuring autonomous motivation (α = 0.84) (“For the pleasure I feel when I’m doing physical exercise”), three items measuring integrated motivation (α = 0.94) (“Because physical activity is an integral part of the lifestyle I chose”), and three items measuring identified motivation (α = 0.91) (“Because I think that physical exercise is good for my personal development”). Controlled motivation score was calculated including three items measuring introjected motivation (α = 0.77) (“Because I would feel bad if I did not make this effort”), and three items measuring external motivation (α = 0.80) (“To avoid having to hear the reproaches of certain people”). Amotivation score was calculated with three items (α = 0.82) (“I have no idea, I don’t think it helps me”). Responses were given on a 7-point Likert scale ranging from 1 (does not correspond to me at all) to 7 (strongly corresponds to me).

#### Positive and Negative Affective States

The 20-item French version of the Positive and Negative Affect Schedule (PANAS, Watson et al., 1988) was used to measure positive affective states (10 items, e.g., interested, excited, attentive, proud, and active) and negative affective states (10 items, e.g., scared, angry, irritable, jittery, and nervous). Participants were asked to rate the intensity of each adjective on a scale of 1 (not at all or very slightly) to 5 (extremely). The Cronbach alphas in this study were 0.83 for the 10 items assessing positive affective states, and 0.76 for the 10 items measuring negative affective states.

#### Apathy

We used the French patient version of apathy inventory to assess apathy (Robert et al., 2002). It evaluates three dimensions that are emotional blunting (e.g., “Do you have the impression that you are as affectionate as usual?,” “Do you express your emotions?”); lack of initiative (e.g., “Do you spontaneously initiate a conversation?,” “Do you take decisions and initiatives?”); and lack of interest (e.g., “Do you take an interest in what is happening around you, your future life?,” “Are you interested in your friends and family members?,” “Are you as enthusiastic about your interests as before?”). The score for each dimension is obtained by using a scale ranging from 1 (weak emotional blunting, weak loss of initiative, weak loss of interest) to 12 (severe emotional blunting, severe loss of initiative, severe loss of interest). In accordance with Robert et al. (2002), we made a global assessment of apathy. In this study, Cronbach’s alpha was 0.76.

#### Boredom

We used the French version of the Boredom Proneness Scale (Gana and Akremi, 1998). This 28-item questionnaire comprises statements such as “I often find myself with nothing to do, with time on my hands.” True/false responses are given depending on whether patients recognize themselves in the statements. A high score indicates a strong inclination to boredom. In this study, Cronbach’s alpha was 0.76.

### Procedure

Permission to conduct this study was granted by the University’s Human Research Ethics committee, File no. 2017-11-02—recommendation of the Ethics Committee for Non-Interventional Research Tours-Poitiers (CERNI-TP). A-CRS were contacted and gave their permission to conduct this study in their institutions. Older people in these services were contacted and only those who volunteered and met the criteria were included in the study. They were fully informed of the study procedures and their rights. Written informed consent was completed by all participants, and participants responded individually and anonymously to a 90 min comprehensive questionnaire including all self-report questionnaires in their room. The procedure was discussed between all authors to ensure standardization, and for consistency, all authors participated in a training session. No rewards/compensation was received by participants.

### Data Analysis

To test our hypotheses, we used partial least squares path modeling (PLS-PM), which is a variance-based structural equation modeling technique that does not rely on distributional assumptions and is able to deal with small sample sizes and non-normality. We used boot-strapping with 100 resamples to estimate probability values for significance testing. All PLS-PM analyses were conducted using the R package PLS-PM (Sanchez, 2013). To retain a reasonable number of manifest variables in the model, each construct was measured by parcels or aggregates of items. According to Little et al. (2002) we used a domain-representative approach for autonomous and controlled motivation for physical activities and apathy. These authors indicated that this choice limited several problems including high instability and unacceptable parameter estimates. Thus, rather than forming parcels using the different types of motivation for physical activities or the different sub-dimensions of apathy, the first, second, and third items of each motivation subscale (intrinsic, integrated, and identified motivations for autonomous motivation; introjected and external motivations for controlled motivation; emotional blunting, lack of initiative, and lack of interest) were averaged to create three parcels. For constructs pertaining to basic psychological need satisfaction, boredom, and positive and negative affective states, parcels were created using a random assignment approach. Finally, for amotivation, supportive/controlled environment, and causality orientations, items were used as manifest variables. As each causal subsystem sequence of paths is estimated separately in the PLS-PM approach, a rule of thumb for PLS-PM estimations suggests that the sample size should be equal to the larger of the following (Tenenhaus et al., 2005): (1) 10 times the number of indicators of the scale with the largest number of manifest indicators, or (2) 10 times the largest number of structural paths directed at a particular construct in the inner path model. As such, the sample size of the current study was acceptable for conducting PLS-PM analysis.

To determine the sources of poor overall model fit, we used a two-step modeling approach. First, the outer (measurement) model allowed us to focus on the factor structure underlying the items and/or parcels of each construct to examine the psychometric properties of each of the constructs. Secondly, the inner model consisted of simultaneously testing the structural and measurement models and allowed us to focus on conceptual connections among the latent factors.

## Results

Descriptive statistics and the correlation matrix are shown in Table 3. The results of the psychometric properties of the PLS-PM measurement model (outer model) are summarized in Table 4. According to Sanchez (2013) were used several psychometric indicators to assess the quality of the measurement model): (a) standardized factor loadings, (b) composite reliability values (ρ), (c) average variance extracted (AVE) values, and (d) an eigenvalue analysis of the correlation matrix of each set of manifest variables. In PLS-PM, the loadings are correlations between a latent variable and its manifest variables (Sanchez, 2013). Loadings greater than 0.70 are generally considered as acceptable. *ρ*-values, which focus on the variance of the sum of manifest variables in the latent variable of interest, measure the overall reliability of a collection of heterogeneous but similar manifest variables (Sanchez, 2013). A value of 0.70 or greater indicates good reliability (Raykov, 2001). Average variance extracted values describe the variance captured by measurement errors as opposed to the variance attributable to the latent factors. A value of 0.50 or greater indicates good reliability, as the variance of the construct is greater than the error variance. Eigen analysis of the correlation matrix is based on the size of the first eigenvalue. If a block (of manifest variables) is unidimensional, then the first eigenvalue should be larger than 1 and the second eigenvalue should be smaller than 1 (Sanchez, 2013). The results provided strong evidence for the reliability and validity of all the constructs examined in the current study, as indicated by the loadings, *ρ*-values, AVE values, and first and second eigenvalues reported in Table 4.

**TABLE 3:** Descriptive statistics and correlations.

**Variables**		***M***	***SD***	**A**			**B**						**C**			**D**	**E**	**F**		**G**		
				**1**	**2**	**3**	**4**	**5**	**6**	**7**	**8**	**9**	**10**	**11**	**12**			**15**	**16**	**17**	**18**	**19**
A. motivation toward health-oriented physical activity	1. Autonomous	4.98	1.69	–																		
	2. Controlled	2.80	1.09	0.43***	–																	
	3. Amotivation	1.58	1.19	-0.45***	0.05	–																
B. Interpersonal behaviors	4. Autonomy support	4.39	1.40	0.34***	0.29***	-0.20*	–															
	5. Control of autonomy	2.34	1.49	-0.15	0.08	0.32***	-0.38***	–														
	6. Competence support	4.96	1.16	0.25**	0.25**	-0.22**	0.71***	-0.30***	–													
	7. Control of competence	1.43	0.83	-0.06	0.30***	0.40***	0.01	0.44***	-0.12	–												
	8. Relatedness support	3.67	1.54	0.18*	0.18*	-0.05	0.57***	-0.18*	0.55***	0.18*	–											
	9. Control of relatedness	2.63	0.93	-0.03	0.15	0.13	-0.16	0.31***	-0.21*	0.34***	-0.33***	–										
C. General causality orientations	10. Autonomy	31.13	8.89	0.16	-0.09	-0.15	0.23**	-0.30***	0.25**	-0.11	0.19*	-0.17*	–									
	11. Control	31.82	7.33	-0.06	0.06	-0.01	-0.16*	0.13	-0.09	-0.08	-0.17*	0.12	-0.43***	–								
	12. Impersonal	13.01	6.75	-0.16*	0.25**	0.34***	-0.08	0.35***	-0.17*	0.61***	0.06	0.19*	-0.31***	-0.21*	–							
D. Boredom		10.27	4.88	−0.25**	0.08	0.29***	-0.21*	0.26**	-0.18*	0.20*	-0.12	0.05	-0.49***	0.15	0.23**	–						
E. Apathy		5.03	5.67	−0.23**	-0.19*	0.06	-0.43***	0.26**	-0.46***	0.00	-0.36***	0.14	-0.31***	-0.01	0.10	0.39***	–					
F. Affects	15. Positive	27.17	8.04	0.16	0.08	-0.08	0.39***	-0.16	0.28***	-0.00	0.26**	0.01	0.40***	-0.20*	-0.12	-0.49***	-0.42***	–				
	16. Negative	17.89	6.58	-0.13	0.16*	0.10	-0.12	0.25**	-0.06	0.30***	0.02	0.13	-0.20*	0.09	0.21**	0.37***	0.21**	-0.07	–			
G. Basic psychological needs	17. Autonomy	15.95	4.57	0.23**	0.21*	-0.12	0.56***	-0.26**	0.49***	-0.01	0.40***	-0.06	0.19*	-0.08	-0.09	-0.23**	-0.47***	0.41***	-0.14	–		
	18. Competence	17.35	3.99	0.25**	0.05	-0.13	0.44***	-0.23**	0.38***	-0.13	0.37***	-0.12	0.28***	-0.10	-0.24**	-0.39***	-0.41***	0.48***	-0.18*	0.66***	–	
	19. Relatedness	17.78	3.62	0.02	0.21*	-0.02	0.32***	-0.15	0.37***	0.14	0.42***	-0.17*	0.13	-0.15	0.14	-0.09	-0.37***	0.20*	0.01	0.37***	0.26**	–

N = 146. *p < 0.05; **p < 0.01; ***p < 0.001.

**TABLE 4:** Psychometric properties.

**Scale**	**Constructs (latent variables)**	**Construct level statistics**	**Parcels**	**SFL**
General	Autonomy	λ_*1*_ = 1.82; λ_*2*_ = 0.69	1	0.74
causality		ρ = 0.82; AVE = 0.60	2	0.81
orientations			3	0.76
	Control	λ_*1*_ = 1.45; λ_*2*_ = 0.89	1	0.67
		ρ = 0.73; AVE = 0.48	2	0.77
			3	0.64
	Impersonal	λ_*1*_ = 1.99; λ_*2*_ = 0.64	1	0.84
		ρ = 0.85; AVE = 0.66	2	0.81
			3	0.79
Interpersonal behaviors	Autonomy support	λ_*1*_ = 2.47; λ_*2*_ = 0.69	1	0.72
			2	0.81
		ρ = 0.86; AVE = 0.62	3	0.79
			4	0.82
	Competence support	λ_*1*_ = 2.64; λ_*2*_ = 0.58	1	0.83
			2	0.79
		ρ = 0.80; AVE = 0.66	3	0.83
			4	0.79
	Relatedness support	λ_*1*_ = 2.07; λ_*2*_ = 0.82	1	0.74
			2	0.60
		ρ = 0.85; AVE = 0.52	3	0.80
			4	0.71
	Controlled autonomy	λ_*1*_ = 2.77; λ_*2*_ = 0.55	1	0.88
			2	0.85
		ρ = 0.88; AVE = 0.69	3	0.82
			4	0.78
	Controlled competence	λ_*1*_ = 2.40; λ_*2*_ = 0.69	1	0.68
			2	0.72
		ρ = 0.90; AVE = 0.59	3	0.82
			4	0.85
	Controlled relatedness	λ_*1*_ = 1.97; λ_*2*_ = 0.82	1	0.64
			2	0.64
		ρ = 0.79; AVE = 0.48	3	0.82
			4	0.67
Basic	Autonomy	λ_*1*_ = 2.20; λ_*2*_ = 0.45	1	0.85
psychological		ρ = 0.89; AVE = 0.73	2	0.88
needs			3	0.84
	Competence	λ_*1*_ = 1.93; λ_*2*_ = 0.58	1	0.76
		ρ = 0.84; AVE = 0.64	2	0.82
			3	0.82
	Relatedness	λ_*1*_ = 2.14; λ_*2*_ = 0.63	1	0.89
		ρ = 0.88; AVE = 0.71	2	0.90
			3	0.73
Motivation	Autonomous	λ_*1*_ = 2.78; λ_*2*_ = 0.15	1	0.85
toward		ρ = 0.97; AVE = 0.93	2	0.96
health-			3	0.97
oriented	Controlled	λ_*1*_ = 2.07; λ_*2*_ = 0.50	1	0.84
physical		ρ = 0.86; AVE = 0.68	2	0.76
activity			3	0.87
	Amotivation	λ_*1*_ = 2.22; λ_*2*_ = 0.49	1	0.86
		ρ = 0.89; AVE = 0.74	2	0.89
			3	0.83
Boredom		λ_*1*_ = 2.28; λ_*2*_ = 0.76	1	0.83
			2	0.78
		ρ = 0.83; AVE = 0.55	3	0.81
			4	0.51
Apathy		λ_*1*_ = 1.61; λ_*2*_ = 0.70	1	0.81
		ρ = 0.77; AVE = 0.53	2	0.67
			3	0.70
Affects	Positive	λ_*1*_ = 2.23; λ_*2*_ = 0.44	1	0.78
		ρ = 0.89; AVE = 0.74	2	0.88
			3	0.91
	Negative	λ_*1*_ = 2.06; λ_*2*_ = 0.54	1	0.73
		ρ = 0.86; AVE = 0.68	2	0.86
			3	0.87

*λ_*i*_, ith eigenvalue of the item correlation matrix; ρ, composite reliability; AVE, average variance extracted; SFL, standardized factor loadings. All SFLs were significant at p < 0.05.*

The quality of the measurement model having been proved the next step was to assess the structural part of the PLS-PM. The largest effects were observed for impersonal orientation (-0.20 ≥ β≥0.66). Autonomy causality orientation significantly and positively predicted the perception of autonomy (β = 0.18), competence (β = 0.16), and relatedness support (β = 0.19), and negatively predicted the perception of autonomy control (β = −0.14). Impersonal orientation significantly and positively predicted the perception of autonomy (β = 0.32), competence (β = 0.66), and relatedness control (β = 0.39), and negatively predicted the perception of competence support (β = −0.20).

Autonomy support significantly and positively predicted autonomy (β = 0.34), and competence needs satisfaction (β = 0.26). Competence support significantly and positively predicted autonomy (β = 0.14), and relatedness needs satisfaction (β = 0.21). Relatedness support significantly and positively predicted autonomy (β = 0.12), competence (β = 0.23) and relatedness needs satisfaction (β = 0.20). Competence control significantly and negatively predicted competence (β = −0.22), and relatedness needs satisfaction (β = −0.30). Relatedness control significantly and negatively predicted relatedness need satisfaction (β = -0.30).

Autonomy need satisfaction significantly and positively predicted controlled motivation (β = 0.25). Competence need satisfaction significantly and positively predicted autonomous motivation (β = 0.20).

**FIGURE 2:**
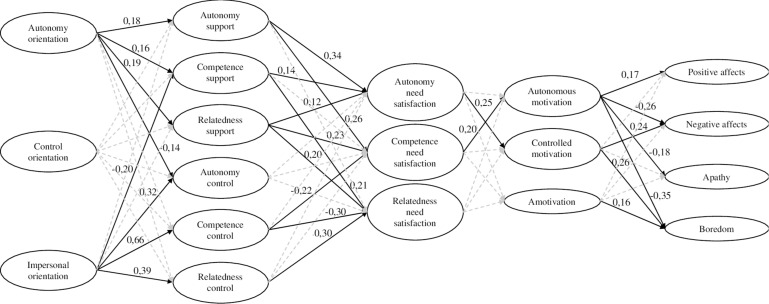
Final model.

Autonomous motivation significantly and positively predicted positive affective states (β = 0.17), and negatively predicted boredom (β = −0.35), apathy (β = −0.18) and negative affective states (β = −0.26). Controlled motivation significantly and positively predicted boredom (β = 0.26) and negative affective states (β = 0.24). Finally, amotivation significantly and positively predicted boredom (β = 0.16). See Figure 2.

## Discussion

The purpose of the current study was to test an integrative model of the role of causality orientations, and a supportive/controlled environment on basic need satisfaction, motivation for health oriented physical activity and emotions in hospital units using the PLS-PM approach. The findings show the SDT was a suitable framework to gain insight into hospitalized older adults’ motivation and behaviors.

The first hypothesis H_0a_ was to investigate the relation between causality orientations and the perception of supportive/controlled environment. Our results indicated that a strong autonomy orientation of patients was positively associated with the perception of a supportive environment and negatively associated with the perception of controlled autonomy. Conversely, an impersonal orientation of patients was positively associated with the perception of a controlled environment and negatively associated with the perception of competence support. Consistent with Deci and Ryan (1985), this first hypothesis showed that the strength of patients’ causality orientations could explain a significant amount of their perception of their environment. Olesen (2011) indicated that individuals who had a strong autonomy orientation were expected to display more curious and receptive attitudes toward others. Applied in the health context, it could be argued that these individuals with an autonomy orientation seek opportunities for self-determination and choice and perceive feedback and strategies provided by health professionals as supportive. Regarding other results, no relationships were found between a controlled orientation and environment in this study. This result is not unique. It was also found in Halvari et al.’s (2017) study and would be due to the low internal consistency of the subscale. Only for some patients does impersonal orientation indicate that they believe that their behavior is initiated and regulated by impersonal forces rather than personal intentions. For them, their environment did not particularly provide the opportunity to support the need for competence (Hagger et al., 2015).

The second hypothesis H_0b_ was to investigate the relationships between the perception of a supportive/controlled environment and the satisfaction of the three basic needs of autonomy, competence, and relatedness. The results of the present study indicated that a perceived supportive environment was associated with need satisfaction. This is in line with numerous SDT studies conducted in various domains and in the health context, Chen et al. (2017) showed that haemodialysis patients experienced greater basic need satisfaction and their health-related quality of life improved, due to the perception of a supportive environment, direction of improvement and clear guidelines provided by their physicians and nurses. Our study also highlighted that controlled competence and relatedness need were negatively associated with competence and relatedness need satisfaction, respectively. This suggests that there is a need for a better understanding of how an environment and particularly its structure which refers to perceived associations between how one behaves and what the result of behaviors is going to be fosters both competence and relatedness need satisfaction and imposes control which undermines this satisfaction.

The third hypothesis was to investigate whether basic needs satisfaction was positively associated with autonomous motivation for health (H_0c_). This result is consistent with other studies (e.g., Kirkland et al., 2011; Ng et al., 2012). However, surprisingly, we found a positive relationship between autonomy need satisfaction and controlled motivation. This could be explained by the fact that participants of 65 years and older want to be guided, advised, and encouraged by professionals, especially when carrying out a physical activity (Rodriguez et al., 2008). Participants follow the advice of professionals to achieve the desired objectives and perform the rehabilitation activities as instructed, and not for their own reasons. Blanchard et al. (1988) showed that patients wanted to be given medical and non-medical information, but that they preferred their physicians to make decisions. Our results suggest that autonomous motivation and controlled motivation coexist in healthcare, particularly in rehabilitation. One plausible explanation for this could be that to perceive themselves as competent and to maintain high levels of autonomous motivation, patients seek to achieve the performance standards set by professionals.

The last hypothesis was to highlight relationships between the three forms of motivation (Deci and Ryan, 2000) and emotions H_0d_. Our results showed that autonomous motivation was positively linked to positive affective states and negatively linked to negative affective states, apathy, and boredom. Conversely, controlled motivation and amotivation were, respectively, associated with negative affective states and boredom. These results are congruent with SDT studies (e.g., Deci and Ryan, 2000), and suggest that careful attention is needed for tasks that are relatively boring (e.g., Van Hooft and Van Hooft, 2018).

In sum, the integrative model shows that autonomy orientation positively associated with the perception of a supportive environment was related to need satisfaction, autonomous motivation for health-oriented physical activity and high scores on positive affective states. Conversely impersonal orientation positively associated with the perception of a controlled environment was related to undermining need satisfaction, controlled motivation for health-oriented physical activity and amotivation and high scores on both negative affective states and boredom. This study suggests that focusing on causality orientations related to a specific context such as A-CRS services may help health professionals. We found that this result was particularly important because if Ntoumanis et al. (2020, p. 3) in their meta-analysis of 73 SDT intervention studies in health context found that “SDT interventions have focused on enhancing perceptions of need support, often by training significant others to utilize behaviors that facilitate experiences of psychological need satisfaction and foster autonomous motivation for behavioral engagement,” the present study shows that creating a supportive environment is a key point, but that it is also necessary to consider the autonomous orientation that allows individuals to be motivated by engagement and inherent interest in activities, focusing on how they could act as their own agent, or on how activities could deepen their experiences.

From a practical perspective, there is an opportunity for health professionals to allow patients to express their point of view on arrival in addition to reading their medical records, to listen to how they perceive things and question them as often as possible for understanding his/her perspective. This encourages patients to adopt an autonomous orientation, related to the perception that health professionals are supportive and involved, which enables them to satisfy their three basic needs, to improve acceptance of professionals’ recommendations and to adopt positive affective states during exercise (Kwan et al., 2011). In this study, patients who had an impersonal orientation tended feel more disengaged from their actions, and our study highlights the following sequence: impersonal orientation—controlled environment—undermining basic need satisfaction, and a positive relationship with controlled motivation or amotivation, negative affective states, and boredom. Cooper et al. (2015) indicated that this orientation tended to develop when there were few opportunities in the environment for autonomy, or when there were few rewarding stimuli and challenging tasks.

## Limitations

This study has several limitations. The first limitation may be the use of self-report questionnaires. Because participants were hospitalized on site, their use appeared to be the most effective and acceptable form of evaluation to be implemented. However, behavior is multi-determined and the general scale of causal orientations lacks sufficient specificity to capture much variance among all determinants (Deci and Ryan, 1985). Observer-rated measures should complement this scale to provide more information on causality orientations. A second limitation concerns the fact that data are limited to a targeted sample and could be not generalizable to the hospitalized older adults’ population. Moreover, among the 146 patients who completed all measures, 75% came from one A-CRS. There is therefore a difference between the center with a high response rate and the others. This fact, combined with the fact that our sample consisted of volunteers, means that our results must be taken with caution. A third limitation concerns the choice of a cross-sectional design. Causal inference cannot be made from the data. Finally, if PLS-SEM or SEM seem a useful tool for a better understanding of the validity of health outcomes measures, other alternative models are available in SDT studies highlighting autonomy-supportive approaches. The choice of one model over another will depend on the complexity of the variables and the objectives of the study.

## Conclusion

To conclude, Deci and Ryan (1985) indicated that “causality orientations represent qualitatively different perspectives on the initiation and regulation of behavior” (p. 114), and the present study provides evidence that autonomy orientation promotes actions as self-initiated and volitional, while interpersonal orientation refers to a lack of internalized self-regulation such as experiencing behavior and choices as inefficient, incomprehensible and beyond intentional control. Our study suggests that causality orientations viewed in terms of dispositional motivational orientations can influence and can be influenced by contingencies in psychosocial contexts (e.g., Hodgins et al., 2006). Although some authors have highlighted the interactive effects of causality orientations and a supportive/controlled environment on autonomous motivation (e.g., Deci and Ryan, 2000; Hagger and Chatzisarantis, 2011), others have suggested that their effect could be additive rather than interactive (Williams et al., 1996; Hagger et al., 2015). If more research on this point is needed, our study suggests the importance of better understanding the relationship between motivational dynamics and need satisfaction. Recent meta-analyses (e.g., Gillison et al., 2019; Ntoumanis et al., 2020) have analyzed SDT based interventions promoting motivation and need satisfaction for better understanding health behavior changes. Results showed that most of these interventions resulted in changes in autonomy support, autonomy, competence, and relatedness satisfaction and autonomous motivation, and had a positive impact on health behaviors. Ntoumanis et al. (2020) have also indicated that changes in autonomous motivation and perception of need support were associated with positive changes in health behaviors both at the end of the intervention and at follow up. It is an interesting finding confirming SDT’s views about the beneficial outcomes of need support and autonomous motivation. The present research, in turn, shows the links between types of dispositional causality orientations and supportive/controlled environment that affect the quality of motivation and need satisfaction. Further research is needed to further explore these linkages.

## Data Availability Statement

The original contributions presented in the study are included in the article/supplementary material, further inquiries can be directed to the corresponding author/s.

## Ethics Statement

The protocol was approved by the Human Research Ethics Committee, university of Tours, France, File n° 2017-11-02 – recommendation of the Ethics Committee for Non-Interventional Research Tours-Poitiers (CERNI-TP). Written informed consent were obtained from all subjects in accordance with the Declaration of Helsinki.

## Author Contributions

GS and CF contributed to the conception and design of study and wrote the draft of the manuscript. GM performed the statistical analysis. GS, DA, CG, and CF collected the data. GM, DA, and CG contributed to the manuscript and carried out the corrections in the article. All authors contributed to the article and approved the submitted version.

## Conflict of Interest

The authors declare that the research was conducted in the absence of any commercial or financial relationships that could be construed as a potential conflict of interest.
